# Supplementary material for: Comparison of the Effects of Brazil Nut Oil and Soybean Oil on the Cardiometabolic Parameters of Patients with Metabolic Syndrome: A Randomized Trial
Source: Nutrients. 2019 Dec 23;12(1):46. doi: 10.3390/nu12010046 (PMC7019763; doi:10.3390/nu12010046)
Supplement: Supplementary file 1 [file nutrients-12-00046-s001.pdf]

Table S1. Study data.

Group	Age in years	Gender	BMI T0	BMI T1	WC T0	WC T1	%BF T0	%BF T1	SBP T0	DBP T0	SBP T1	DBP T1	Glucose T0	Glucose T1	TEAC T0	TEAC T1	TBARS T0	TBARS T1	TC T0	TC T1	HDL T0	HDL T1	LDL T0	LDL T1	TG T0	TG T1
BNO	36	M	39.27	38.97	118	118	39.9	39.2	156	108	154	105	274.8	294.8	3.440	2.270	3.796	2.176	179.15	175.56	42.91	33.04	94.34	35.09	209.48	537.16
BNO	63	F	34.68	33.74	115	111	49.6	50.7	95	68	95	66	238.0	242.7	3.374	3.521	2.870	4.028	252.65	315.04	27.97	30.38	169.36	225.71	276.56	294.76
BNO	62	M	30.00	29.93	106	107	28.7	28.5	123	80	126	81	104.2	105.5	3.625	3.536	2.639	2.407	188.21	294.53	36.71	28.35	120.47	206.23	155.11	299.75
BNO	62	F	23.39	23.07	93	92	36.5	37	130	85	128	85	83.7	126.3	3.421	3.647	4.954	2.870	194.87	250.60	48.10	25.95	106.22	179.41	202.74	226.18
BNO	60	F	32.69	32.69	111	111	44.9	47.9	139	70	131	69	93.6	123.9	3.321	3.486	2.176	2.176	209.57	273.50	26.20	25.95	133.64	188.35	248.63	296.01
BNO	60	M	30.71	31.28	99	99	30.5	30.9	141	96	141	85	65.1	78.7	3.190	3.464	5.185	2.407	374.19	253.50	32.03	24.56	300.62	171.24	207.73	288.53
BNO	63	F	40.16	39.64	130	125	51.3	51.2	119	66	144	70	169.5	157.3	3.443	3.472	3.333	1.713	218.12	227.35	38.48	25.19	132.31	155.78	236.66	231.92
BNO	61	F	33.24	31.96	116	110	44.3	43.2	137	79	133	82	216.6	136.3	3.432	1.068	2.407	3.333	245.98	254.53	43.92	31.52	159.07	162.81	214.96	301.00
BNO	47	M	33.09	29.96	118	114	23.9	22.5	179	87	147	87	52.9	100.5	3.412	3.474	2.639	2.176	177.95	139.66	30.00	28.73	104.51	48.43	217.21	312.47
BNO	47	F	31.32	30.79	104	107	39.9	41.8	118	78	120	84	81.2	91.0	3.478	3.570	2.870	2.870	323.93	250.60	38.35	32.78	241.54	164.90	220.20	264.59
BNO	60	F	26.62	27.07	94	96	37.4	36.4	110	71	114	67	278.4	177.3	3.381	3.467	3.333	0.324*	250.26	24291	41.27	43.80	157.62	135.22	256.86	319.45
BNO	64	F	30.61	26.88	101	102	37.8	39	132	73	130	72	150.4	148.8	3.368	3.417	2.639	1.250	189.91	146.84	19.49	14.68	124.64	71.31	228.93	304.24
BNO	63	F	31.84	32.34	109	105	49.3	49.2	139	81	155	80	214.4	244.8	3.445	3.602	4.491	36.898*	236.58	259.32	44.56	19.49	143.40	184.71	243.14	275.56
BNO	63	F	28.56	28.24	109	108	36.7	36.4	122	63	108	68	86.7	91.7	0.322*	3.574	2.407	2.639	325.13	206.15	33.16	34.43	235.85	121.55	280.55	250.87
BNO	64	F	36.12	35.76	120	118	47.3	50.5	143	77	136	77	192.2	244.3	3.375	3.424	2.407	1.481	280.17	310.60	44.30	46.46	195.62	218.86	201.25	226.43
SO	60	F	28.24	28.29	104	101	37.9	37.5	153	80	113	62	107.8	108.5	3.113	3.421	3.565	29.954*	197.09	228.89	45.70	38.61	112.35	147.24	195.26	215.21
SO	62	F	30.68	30.95	108	104	39.2	41	156	80	137	72	130.7	109.5	3.413	3.385	4.259	8.194	237.09	188.89	45.19	62.53	151.65	80.82	201.25	227.68
SO	63	F	24.75	24.12	90	90	37.4	40.2	149	80	124	66	68.1	122.7	3.443	3.579	2.870	1.250	280.17	281.03	35.44	43.04	200.19	195.94	222.69	210.22
SO	64	F	32.45	32.10	109	104	48.6	49.8	106	69	115	74	103.9	100.5	3.312	3.488	2.870	1.944	234.53	275.38	54.43	77.22	141.40	156.27	193.52	209.48
SO	43	F	35.64	35.45	105	100	47.8	48.6	119	81	105	67	205.5	98.3	3.394	3.571	2.407	4.259	251.11	218.80	28.99	35.32	174.99	133.36	235.66	250.62

SO	56	F	30.18	30.09	109	109	43.5	46.6	134	71	122	67	96.1	84.1	3.214	3.535	2.639	1.250	187.69	265.64	47.34	48.86	98.70	181.37	208.23	177.06
SO	54	F	38.05	38.70	117	117	53.5	52.8	146	94	148	103	106.8	110.0	3.346	3.533	3.102	4.259	232.99	254.87	19.11	23.80	162.01	185.49	259.35	227.93
SO	63	F	28.18	27.91	102	96	42.4	42.9	154	80	125	75	111.6	94.1	3.438	3.477	2.407	1.250	217.09	280.34	64.05	45.95	115.94	194.79	185.54	198.00
SO	65	F	34.01	33.60	106	111	51.5	50.1	139	88	135	73	127.7	101.2	3.465	3.463	2.870	3.796	133.85	232.31	47.97	46.96	53.35	141.75	162.59	217.96
SO	51	F	25.16	25.26	87	90	40.1	41.4	148	88	134	81	126.6	101.9	3.523	3.595	3.333	1.713	254.53	310.43	40.00	32.15	152.73	208.95	308.98	346.63
SO	60	F	28.74	28.60	93	90	43.1	43.4	114	71	137	80	103.0	93.6	3.454	3.336	3.102	2.407	218.80	225.81	21.65	19.62	156.26	154.52	204.49	258.35
SO	56	F	39.86	39.61	122	118	52.8	52.4	148	78	137	87	113.6	82.0	3.457	3.595	2.176	5.185	192.14	192.48	39.11	41.65	104.49	119.71	242.64	155.61
SO	61	F	31.42	31.87	101	105	47.2	46.2	133	82	144	84	91.1	91.2	3.475	3.638	3.565	1.713	228.21	252.82	25.44	28.35	166.10	184.67	183.29	199.00
SO	62	F	25.32	24.93	80	84	41.4	43	113	72	99	63	85.0	79.9	3.459	3.263	3.333	2.639	177.26	172.31	38.35	50.38	99.41	90.61	197.51	156.61
SO	57	F	31.68	32.00	102	103	44.9	45.8	120	76	132	90	113.6	188.6	3.447	3.509	2.407	0.787	196.07	204.79	51.90	48.61	96.84	121.52	236.66	173.32
SO	56	M	29.06	28.87	102	99	28.1	26.9	114	76	110	79	162.0	142.2	3.432	3.620	3.796	4.028	226.32	263.59	39.75	36.33	135.41	187.76	255.86	197.51

BNO (Brazil nut oil), SO (Soybean oil), BMI (body mass index, %BF (percent body fat), WC (waist circumference), SBP (systolic blood pressure), DBP (diastolic blood pressure), TC (total cholesterol), LDL (Low-density lipoprotein), HDL (High-density lipoprotein), TG (triglycerides), MDA (malondialdehyde), TEAC (Trolox equivalent antioxidant capacity).*Outliers.
